# Regulation of Pulmonary Vascular Smooth Muscle Contractility in Pulmonary Arterial Hypertension: Implications for Therapy

**DOI:** 10.3389/fphys.2017.00614

**Published:** 2017-08-23

**Authors:** Melissa A. Lyle, Jonathan P. Davis, Frank V. Brozovich

**Affiliations:** ^1^Department of Cardiovascular Diseases, Mayo Clinic Rochester, MN, United States; ^2^Department of Physiology and Cell Biology, Ohio State University Columbus, OH, United States

**Keywords:** contractility, vascular diseases, vascular resistance, signaling pathways, therapeutics

## Abstract

There are two primary components that produce pulmonary arterial hypertension (PAH); aberrant structural changes (smooth muscle cell proliferation, smooth muscle cell hypertrophy, and the deposition of matrix proteins within the media of pulmonary arterial vessels), and excess vasoconstriction. However, in PAH, the target and aim of all current therapeutic agents is to reduce the contractility of the pulmonary vasculature; prostaglandins, phosphodiesterase inhibitors, guanylate cyclase stimulators, endothelin antagonists, NO inhalation and Rho kinase inhibitors all influence signaling pathways in the pulmonary vascular smooth muscle to decrease vasoconstriction, and hence, pulmonary vascular resistance (PVR). This review will therefore primarily focus on discussing the signaling pathways regulating contractility in pulmonary vascular smooth muscle, the mechanism for current treatments, as well as highlighting potential targets for the development of novel therapies.

## Clinical syndrome

Pulmonary hypertension (PH) is defined as a resting mean pulmonary artery pressure (PAP) >25 mmHg. This disease results from progressive changes in the pulmonary vascular bed that increase pulmonary artery pressures, which ultimately leads to right ventricular (RV) failure. This broad diagnosis includes patients not only with intrinsic pulmonary disease, but also those with elevated pulmonary pressures related to left ventricular disease and high output heart failure. The World Health Organization classifies PH into five categories (Simonneau et al., 2013): Category 1 or pulmonary arterial hypertension (PAH); Category 2 or PH associated with left-sided heart disease; Category 3 or PH associated with lung disease or hypoxia; Category 4 or chronic thromboembolic pulmonary hypertension (CTEPH); and Category 5 or PH due to a miscellaneous etiology. Category 1 includes PH due to idiopathic PAH, connective tissue disease, congenital heart disease, pulmonary venoocclusive disease, and pulmonary capillary hemangiomatosis (Barst et al., 2004; McGoon et al., 2004; Archer et al., 2010; McLaughlin et al., 2015). Normal resting mean pulmonary arterial pressure (PAP), pulmonary vascular resistance (PVR) and pulmonary capillary wedge pressure (PCW) are 9–18 mmHg, <3 Wood Units, and 10–12 mmHg, respectively; and this review will focus on idiopathic PAH, which is defined by a resting PAP > 25 mmHg, PVR > 3 Wood units, and PCWP < 15 mmHg (Barst et al., 2004).

Death rates related to PH of any etiology are estimated to be 5.5 per 100,000 for women and 5.4 per 100,000 for men (Hyduk et al., 2005). The prevalence of PAH is difficult to assess, but women of child-bearing age are most frequently affected. Specific disease risk factors for PAH include HIV, sickle cell disease, and schistosomiasis, underscoring the assumption that PAH is an under-diagnosed disease on a global level (Butrous et al., 2008). Despite advancements in therapy, the 1-year incident mortality rate of PAH remains high at 15% (Archer et al., 2010). Prognosis associated with PAH often depends on the existing co-morbidities, and it has been shown that patients with congenital heart disease often have better outcomes than patients with idiopathic PAH (Hopkins et al., 1996).

Clinicians should recognize common symptoms associated with PH, and these include shortness of breath, exertional dyspnea, fatigue, peripheral edema, and early satiety with abdominal distention (Barst et al., 2004). It is also important to identify those patients at increased risk for PAH. Individuals thought to be at higher risk would include any patient with a first degree relative with idiopathic PAH, a genetic-mutation associated with PAH (i.e., BMP4), an underlying connective tissue disorder (i.e., scleroderma), known congenital heart disease, or HIV infection. The physical exam is an important tool to further investigate PAH as a differential diagnosis. Physical exam findings consistent with elevated pulmonary pressures include a RV parasternal lift or heave, jugular venous distention with possible prominent V waves if severe tricuspid regurgitation is present, an accentuated pulmonic component of S2, a diastolic murmur of pulmonary regurgitation, and peripheral edema (Barst et al., 2004), As PH severity increases, an early systolic click and mid-systolic ejection murmur may be auscultated, in addition to a RV S4 gallop. If PH is suspected, a screening transthoracic Doppler echocardiogram (TTE) is appropriate. TTE estimates pulmonary artery systolic pressure (PASP), which is equal to the right ventricular systolic pressure (RVSP) in the absence of any pulmonary outflow tract obstruction. The regurgitant tricuspid velocity and the estimate of right atrial pressure are utilized to estimate RVSP (RVSP = 4v^2^ + right atrial pressure, where v is the velocity of the TR jet in m/s, Ommen et al., 2000). Mild PH is usually defined as a RVSP of 36–50 mmHg, or a resting tricuspid regurgitant velocity of 2.8–3.4 m/s. Assessment of the RV by echocardiography helps to risk stratify patients; however, it is important to emphasize that outcomes are not based solely on pulmonary artery pressures (Kane et al., 2011). When assessing severity of PH by objective measures, RV enlargement and dysfunction, severe tricuspid regurgitation, decrease in cardiac output, and the presence of a pericardial effusion all indicate increased severity and poorer prognosis. Patients with PH can also be separated into those who are pre-symptomatic, those who are symptomatic but compensated, and finally those who are symptomatic but decompensated. For example, patients who are symptomatic but compensated may exhibit shortness of breath and dyspnea on exertion, but they may not have any objective or overt findings of RV failure such as lower extremity edema, hepatic congestion, or syncope, which are signs of RV failure and decompensation.

Once elevated pulmonary pressures have been detected on TTE, other tests are necessary to help delineate and categorize the type of PH. These tests include pulmonary function testing to evaluate for underlying lung disease, screening overnight oximetry to assess for obstructive sleep apnea, ventilation-perfusion lung scintigraphy to rule out chronic thromboembolic PH, and a laboratory evaluation to screen for HIV, connective tissue disease, and underlying liver dysfunction. In addition, the assessment of overall exercise capacity with either cardiopulmonary exercise testing or a 6 min walk test is a vital component of the overall evaluation for PH (Barst et al., 2004).

Right heart catheterization remains the gold standard to confirm the presence of PH, and measurements of PVR and PCW establish the diagnosis of PAH. For patients with PAH, right heart catheterization should always include a vasodilator challenge, with either nitric oxide or nitroprusside, dependent upon pulmonary capillary wedge pressure, to identify “responders.” Currently, patients in whom the vasodilator produces at least a 10 mmHg decrease in mPAP (with a concurrent mPAP < 40 mmHg) in the setting of a normal cardiac output are considered to have a positive vasodilator challenge and are termed “responders.” These patients have a better long term prognosis and benefit from long-term calcium channel blocker (CCB) therapy (Barst et al., 2004). However, patients with functional class IV symptoms (dyspnea at rest) are less likely to respond than patients with class II or III symptoms (dyspnea with moderate activities (class II) or activities of daily living (class III). Studies have demonstrated that CCBs can improve survival in some patients that are responsive to vasodilatory challenge, but there are no clear clinical or hemodynamic parameters to indicate which patients will either “respond” to the vasodilatory challenge or benefit from long-term CCB therapy (Barst et al., 2004). It is also important to recognize that only 10–20% of patients with PAH illustrate any significant vasodilatory response to nitric oxide (Barst et al., 2004; McGoon et al., 2004).

## Etiology/mechanism producing PAH

### Animal models

Although no animal model completely replicates PAH, the mechanism(s) that produce PAH has been investigated using a number of different models, including chronic hypoxia, hypoxia combined with the vascular endothelial growth factor (VEGF) antagonist SU5146, monocrotaline (MCT), and the BMP4 KO. The chronic hypoxia animal model illustrates that the small pulmonary arterioles, which lack significant smooth muscle cells, are rapidly muscularized after the animal is exposed to hypoxia. As part of this process, there is an increase in cells expressing α-smooth muscle actin (α-SM actin) in the walls of previously non-muscular arterioles. These changes are hypothesized to be caused by differentiation of pericytes, migration of smooth muscle cells, differentiation of local fibroblasts, or transdifferentiation of endothelial cells into mesenchymal-like cells (Stenmark et al., 2009). This process is followed by the thickening of the muscularized precapillary pulmonary arteries. Inflammation is also thought to contribute to the remodeling process of pulmonary arterioles in the setting of chronic hypoxia (Stenmark et al., 2009).

Another animal model uses a single subcutaneous implantation of the VEGF receptor blocker SU5416, semaxinib, combined with hypoxia. Abe et al. (2010) evaluated rats after receiving a single injection of SU4516 in conjunction with 3 weeks of hypoxia. These rats had concentric laminar neointimal proliferation in addition to 2 different patterns of complex plexiform lesions, a late marker of PH, including a complex stalk-like lesion within the blood vessel lumen and an aneurysm-like lesion extending outside the vessel lumen into the lung parenchyma. It is unclear how the addition of a VEGF antagonist to hypoxia produces plexiform lesions. However, this study confirmed that plexiform lesions, despite which type, did not form until several weeks after severe PAH developed, which suggests that plexiform lesions do not produce PAH, but rather develop late in the disease.

### Structural changes

The two main components thought to be responsible for PAH include structural changes within the pulmonary vasculature and excess vasoconstriction of the pulmonary vasculature (Archer et al., 2010). The structural changes include smooth muscle cell proliferation, smooth muscle cell hypertrophy, and the deposition of matrix proteins within the media of the pulmonary arterial vessels (McLaughlin et al., 2015), which will influence the properties of the pulmonary arteries (Table 1). The classic pathophysiologic changes in PAH are described by the Heath Edwards classification: Grade 1, medial hypertrophy; Grade 2, cellular intimal reaction and proliferation; Grade 3, the formation of concentric laminar neointimal lesions; and Grade 4, the development of plexiform lesions.

**Table 1:** Structural changes and mechanical properties.

**Change**	**Contractility/Force**	**PVR**
Smooth muscle proliferation	No change	Increase
Smooth muscle hypertrophy	Increase	Increase
Infiltration and deposition of cells and proteins	No change	Increase

*In PAH, the structural changes that occur within the pulmonary vasculature all influence the mechanical properties of the vessel and result in an increase in PVR*.

The histologic findings of PAH include intimal hyperplasia, medial hypertrophy, adventitial proliferation, thrombosis *in situ*, infiltration of inflammatory cells, and the presence of angioproliferative “plexiform lesions” (Archer et al., 2010). At the level of the media, pulmonary artery smooth muscle cell apoptosis is suppressed, resulting in proliferation, which combined with collagen replacement of intimal cells leads to the classic “onion-skin” appearance of intimal hyperplasia. This suggests that the pathogenesis of PAH is similar to that underlying cancer, given both diseases show excess proliferation and impaired apoptosis. In both PAH and cancer, pyruvate dehydrogenase kinase (PDK) is elevated, and this enzyme is responsible for phosphorylation and inhibition of pyruvate dehydrogenase (PDH), a vital enzyme regulating the rate of oxidative metabolism. PDK activation results in a metabolic shift to glucose metabolism, and these metabolic abnormalities enhance cell proliferation and impair apoptosis (Archer et al., 2010).

Other factors responsible for the unchecked proliferation include the down regulation of Bone Morphogenetic Protein Receptor Type 2 (BMPR2), which emphasizes the existence of a genetic component to PAH. More than 80% of patients with familial PAH have loss-of-function mutations in BMPR2, which results in cell proliferation. BMPR2 is an active serine-threonine kinase receptor, which forms heterodimers with any type 1 receptor (BMPR1A, BMPR1B, Alk 1, and Alk 2) after binding with a ligand to result in phosphorylation of the intracellular portion of the type 1 receptor. Following receptor activation, a SMAD cascade is activated, resulting in Smad4 translocation to the nucleus to regulate gene transcription. Most BMPR2 mutations associated with PAH lead to abnormal SMAD signaling, which suppresses apoptosis resulting in proliferation of vascular cells. BMPR2 mutations are associated with familial or hereditary PAH; however, these mutations are markedly less common in non-familial category 1 PAH. Other genetic factors thought to be associated with PAH include mutations in endoglin (ENG), caveolin 1 (CAV1), potassium channel subfamily K member 3 (KCNK3), and the serine/threonine-protein kinase receptor R3, also referred to as Activin-like kinase type 2 receptor (ALK1; ACVRL1) (Archer et al., 2010).

The plexiform lesion is thought to be the hallmark of end-stage PAH, and is produced by abnormal angiogenesis, which leads to a subsequent increase in vascular resistance. These lesions consist of a network of channels that are lined by endothelial cells and separated by core cells, either myofibroblasts, smooth muscle cells, endothelial cells, or undifferentiated cells (Abe et al., 2010). Classic plexiform lesions are not present in either the chronic hypoxia model or the MCT model. It is hypothesized that chronic hypoxia itself is an insufficient stimulus to develop plexiform lesions; and in the MCT model, animals succumb to cardiac or renal dysfunction prior to the development of plexiform lesions (Abe et al., 2010). It is thought that widespread endothelial apoptosis early in PAH results later in apoptosis-resistant endothelial precursor cells that will eventually form plexiform lesions. Disarrayed endovascular angiogenesis then results from proliferation of phenotypically abnormal cells because of phagocytosis of the apoptotic monolayer endothelial cells, activation of stem cell-like endothelial cells, or attachment of bone marrow-derived cells to the endothelium. Megakaryocytes, mast cells, and dendritic cells can all be released and attach to the matrix, with further growth factors released by megakaryocytes contributing to angiogenesis. The end result of these changes is the development of complex plexiform lesions (Archer et al., 2010).

In addition, pathologic specimens from patients with PAH reveal an increased number of macrophages, T and B lymphocytes, and mast cells, suggestive of an underlying inflammatory process. Cytokines and chemokines are elevated in PAH, and often associated with a worse prognosis (Price et al., 2012). The proinflammatory cytokines, chemokines, growth factors, and serotonin trigger proliferation and migration in smooth muscle cells, endothelial cells, pericytes, and fibroblasts. Further, data are consistent with a role for the immunosuppressant mycophenolate mofetil in the prevention of MCT-induced PAH (Voelkel et al., 2006; Archer et al., 2010). This raises the question of whether the immune system could be a target for PAH therapy.

### PAH and persistent vasoconstriction

**Figure 1:**
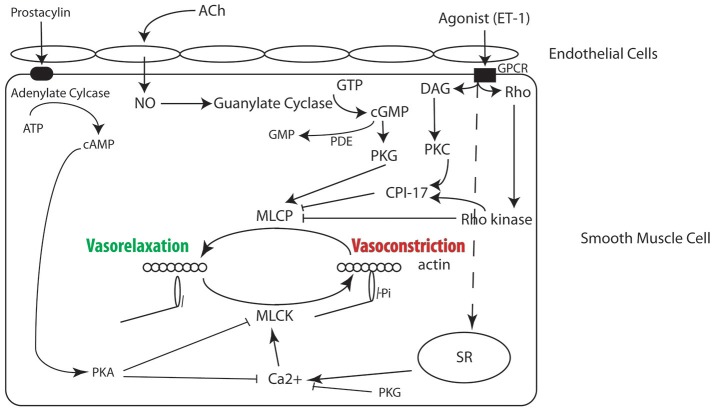
Signaling pathways in smooth muscle regulating contractility, many of which have been implicated in the molecular mechanism for PAH and are the target of therapy (see text for details).

Another component contributing to the pathogenesis of PAH is abnormal smooth muscle cell contractility. This hypercontractility can be caused by exacerbated mechanisms that lead to smooth muscle contraction, but also by blunting mechanisms that would normally produce relaxation of the smooth muscle. For instance, PAH is associated with a decrease in sensitivity to nitric oxide (NO) mediated vaso-relaxation (McGoon et al., 2004). The mechanisms that specifically contribute to the persistent vasoconstriction, the increase in PVR, and the decrease in vasodilatory response to nitric oxide are not well-understood. It has been well-established, however, that VSMCs can modulate their phenotype from a contractile to a non-contractile, proliferative phenotype in many settings, including atherosclerosis, hypertension, or PAH (Griendling and Harrison, 1999; Bennett et al., 2016). It is possible that this phenotypic switch is the underlying mechanism behind the persistent constriction of PAH. However, an increase in contractility of pulmonary vascular smooth muscle cells (VSMCs), producing a higher level of vasoconstriction, also produces PAH (Archer et al., 2010). There are a number of signaling pathways that regulate contractility in smooth muscle (Figure 1); many have been implicated in the pathogenesis of PAH, and will be discussed further in this review.

The crux of persistent vasoconstriction associated with PAH is based on the increased activation of smooth muscle myosin, and which is determined by the balance of the activities of myosin light chain kinase (MLCK) and myosin light chain phosphatase (MLCP) (Gong et al., 1992). MLCK is found in smooth, cardiac, skeletal, and non-muscle cells (Hartshorne et al., 2004). The MYLK1 gene encodes both the non-muscle and smooth muscle isoforms of MLCK, and non-muscle (NM) myosin and smooth muscle (SM) myosin are the only known substrates for MLCK. MLCP is a trimeric enzyme consisting of catalytic subunit, myosin-targeting subunit (MYPT1) and a 20 kDa subunit of unknown function (Hartshorne et al., 1998). Alternative splicing of central and 3' exons produces isoforms of the MYPT1, and isoform expression is developmentally regulated, tissue specific, and modulated in disease (Brozovich et al., 2016). In addition to Ca^2+^ dependent changes in vascular tone, there are also Ca^2+^ independent contractions (Ca^2+^ sensitization) and Ca^2+^ independent relaxation (Ca^2+^ desensitization). Factors affecting these processes include RhoA/Rho-kinase, CPI-17, PKG, and NO/cGMP, which are discussed below (Somlyo and Somlyo, 2003; Brozovich et al., 2016).

### Regulation of contraction in smooth muscle cells

For a complete treatment of this topic we would refer readers to a number of reviews on the regulation of smooth muscle cell contraction (Arner and Pfitzer, 1999; Somlyo and Somlyo, 2003; Brozovich et al., 2016). Briefly, contraction of VSMCs, like all muscle cells, is ultimately regulated by the interaction of myosin with actin (Figure 1), and activation of smooth muscle myosin is regulated by the phosphorylation of the regulatory light chain (RLC) of smooth muscle myosin. RLC phosphorylation is determined by the balance of the activities of two enzymes, MLCK and MLCP (Gong et al., 1992). MLCK is Ca^2+^-dependent; an increase in Ca^2+^ binds to calmodulin, and the Ca^2+^-calmodulin complex activates MLCK, which phosphorylates Ser19 of the RLC (Ikebe and Hartshorne, 1985). The phosphorylation of the RLC activates the smooth muscle myosin ATPase by increasing the rate of product release ~1,000-fold (Sellers and Adelstein, 1985), which increases the interaction of myosin with actin that produces force and/or tone. The RLC is dephosphorylated by MLCP (Hartshorne et al., 1998; Somlyo and Somlyo, 2003), and a decrease in RLC phosphorylation causes vasorelaxation. Therefore, the balance between MLCK and MLCP regulates the level of RLC phosphorylation (or smooth muscle activation) and force/tone in the vasculature (Gong et al., 1992). Although MLCK is regulated by Ca^2+^, MLCP is regulated by a number of other signaling pathways (Figure 1).

Depolarization of the smooth muscle cell results in the opening of voltage dependent L-type Ca^2+^ channels (LTCC) and increase Ca^2+^ flux, which produces an increase in intracellular Ca^2+^ (Brozovich et al., 2016). An increase in Ca^2+^ also occurs with activation of G-protein coupled receptors (Brozovich et al., 2016); agonist activation of G_q/11_ activates phospholipase C, which produces an increase in IP_3_ and diacygylcerol (DAG). IP_3_ binds to the IP_3_ receptor on the SR, which causes Ca^2+^ release, while DAG activates PKC. PKC subsequently phosphorylates CPI-17, which inhibits MLCP. The activation of G_12/13_ results in activation of guanine nucleotide exchange factors (GEFs), and GEFs facilitate the exchange of GDP–bound RhoA for GTP-bound RhoA and the subsequent activation of Rho kinase. Rho kinase phosphorylates the myosin targeting subunit (MYPT1) of MLCP, which inhibits MLCP activity, as well as CPI-17. Thus, both membrane depolarization and agonist activation result in activation of MLCK, while agonist activation also inhibits MLCP, which tips the balance to increase the phosphorylation of the RLC, resulting in force production and/or vasoconstriction.

Several signaling pathways also promote smooth muscle cell relaxation. The NO/PKG signaling pathway not only decreases intracellular Ca^2+^ (decreases the activation of MLCK), but also activates MLCP; both mechanisms result in a decrease in the phosphorylation of the smooth muscle myosin RLC and produce relaxation/vasodilatation (Brozovich et al., 2016). Activation of PKA signaling has also been demonstrated to both decrease intracellular Ca^2+^ and inhibit MLCK (Miller et al., 1983), which also decreases RLC phosphorylation and force. Thus, there are a plethora of signaling pathways that play a role in regulating RLC phosphorylation, and numerous key elements in these signaling pathways are compromised in disease and are pharmaceutical targets for treating PAH.

### Rho kinase

The RhoA/Rho kinase (ROK) pathway is one of the main mediators of smooth muscle Ca^2+^ sensitization (Somlyo and Somlyo, 2003; Brozovich et al., 2016). Rho kinase has been linked to the pathogenesis of PAH, as the inhibition of Rho kinase lowers pulmonary artery pressures in some animal models of PAH (Oka et al., 2007), and treatment with the Rho kinase inhibitor, fausidil, has been clinically approved for PAH (McGoon et al., 2004; Brozovich et al., 2016). RhoA is a small monomeric G-protein, encoded by the gene RHOA, and is a member of the Rho family of GTP-binding proteins. RhoA is activated by the stimulation of G protein coupled receptors (GCPR) by GCPR agonists, such as endothelin-1, thromboxane A2, and serotonin. The active RhoA then translocates from the cytosol to the membrane to interact with its downstream targets including Rho kinases (ROKs). ROKs are serine/threonine kinases, with two isoforms (ROKβ and ROKα). Rho kinase is most frequently activated by RhoA, and can also be activated by increases in Ca^2+^ (Mita et al., 2002).

There are a number of targets of ROK (Brozovich et al., 2016), including a protein kinase C (PKC) mediated myosin phosphatase inhibitor (CP1-17) and the myosin binding unit (MYPT1) of MLCP. Rho kinase phosphorylates both CPI-17 (Thr38) and MYPT1 (Thr696 and Thr853). Phosphorylated CPI-17 binds to the catalytic core of the catalytic subunit of MLC phosphatase, which inhibits the activity of the phosphatase. (Eto, 2009) MYPT1 phosphorylation at either Thr696 (Kitazawa et al., 1991) or Thr853 (Velasco et al., 2002) also inhibits MLC phosphatase activity. Since RLC phosphorylation is determined by the balance of the activities of MLCK and MLCP, Rho kinase mediated inhibition of MLC phosphatase results in an increase in RLC phosphorylation at a constant Ca^2+^ (i.e., Ca^2+^ sensitization; Kitazawa et al., 1991). Thus, inhibiting the RKO pathway with fausidil will promote vasorelaxation.

### NO/PKG

Nitric oxide (NO) is a gas, which diffuses into smooth muscle cells. NO then stimulates soluble guanylate cyclase (sGC), which results in the conversion of guanosine 5'triphosphate (GTP) to cGMP, which will activate type 1 cGMP-dependent serine/threonine protein kinase G (PKG-1). PKG in turn phosphorylates a number of targets that result in smooth muscle relaxation including the maxi K^+^ channel (Alioua et al., 1998), the voltage dependent Ca^2+^ channel (Schmidt et al., 1993), the SR (Twort and van Breemen, 1988), as well as MLCP (Brozovich et al., 2016). PKG phosphorylation of large conductance Ca^2+^ activated K^+^ channels results in membrane hyperpolarization, of Ca^2+^ channels results in a decrease in Ca^2+^ flux, of phospholamban produces an increase in SR Ca^2+^ uptake and of the SR IP_3_ receptor decreases SR Ca^2+^ release, all of which contribute to decreasing intracellular Ca^2+^, which results in smooth muscle relaxation (Lincoln et al., 2001). PKG has also been demonstrated to phosphorylate MYPT1 at Ser695 and Ser852, which inhibits Rho kinase mediated MYPT1 phosphorylation. (Wooldridge et al., 2004; Nakamura et al., 2007) PKG also phosphorylates LZ+ MYPT1 (leucine zipper positive) isoforms at Ser667, which results in a Ca^2+^ independent increase in MLC phosphatase activity (Yuen et al., 2014) and thus, dephosphorylation of the RLC and subsequent vasorelaxation (Chen et al., 2013; Yuen et al., 2014). PKG-1 also phosphorylates RhoA at Ser188 to inhibit its membrane association, and therefore prevents activation of its downstream targets, such as Rho kinase (Ellerbroek et al., 2003).

It has been well-established that interference in the NO/cGMP pathway can result in PH. PKG-1 KO mice have impaired NO/cGMP dependent vasorelaxation, and eventually develop PH (Zhao et al., 2012). A decrease in PKG would decrease the sensitivity to NO mediated (or flow) vasodilatation, and hence increase vascular tone. Zhao et al. illustrated that the PKG-1 deficiency also resulted in activation of RhoA-ROK, causing vascular remodeling, which contributed to vasoconstriction. Ramchandran et al. (2014) illustrated that a selective mutation in the NH2-terminus leucine zipper protein interaction domain of PKG-1, which mediates the interaction of PKG-1 with MYPT1 (Surks et al., 1999), resulted in progressive increases in RV systolic pressure and resultant right ventricular hypertrophy. Although the exact mechanism that produces PH from the loss of PKG-1 has not been entirely elucidated, persistent vasoconstriction would be expected from the loss of nitric oxide mediated vasodilatation.

### Novel mechanisms for PAH

As mentioned, the pulmonary vasculature in PAH is characterized by resting vasoconstriction, in part secondary to a decrease in sensitivity to nitric oxide (NO). MLCP has the myosin-targeting subunit (MYPT1), and PKG-1 activates MLCP. PKG induced activation of MLCP has been demonstrated to be dependent on MYPT1 isoform expression. LZ+ MYPT1 isoforms, but not LZ− MYPT1 isoforms are phosphorylated and subsequently activated by PKG (Yuen et al., 2011, 2014). Similarly, only LZ− MYPT1 isoforms are phosphorylated by Rho kinase (Lin and Brozovich, 2016). Thus, the LZ MYPT1 domain is important for the regulation of MLCP activity, and therefore important for determining the balance between vasoconstriction and vasorelaxation of vascular smooth muscle. In both hypoxia (Singh et al., 2011) and MCT induced PAH (Konik et al., 2013), there is a decrease in relative LZ+ MYPT1 expression. This shift in MYPT1 isoform expression from LZ+ to LZ− MYPT1 would produce both a decrease in sensitivity to NO mediated relaxation (Ca^2+^ desensitization) and an increase in sensitivity to Rho kinase mediated constriction (Ca^2+^ sensitization), which would increase in vascular tone and PVR. Thus, the changes of MYPT1 isoform expression observed in PAH would contribute to the pathogenesis of this disease.

In addition to the changes in LZ+/LZ− MYPT1 isoform expression, Konik et al. (2013) demonstrated that the expression of non-muscle (NM) myosin is significantly increased in MCT induced PAH, and Packer et al. (1998) showed that NM myosin expression also increases in pulmonary smooth muscle during hypoxia. Similar to SM myosin, the NM myosin ATPase is regulated by phosphorylation of its RLC (Cremo et al., 2001); however, the ATPase from NM myosin is much slower than that for smooth muscle myosin (Kovacs et al., 2003; Wang et al., 2003). Therefore, compared to SM myosin, NM myosin's attachment time to actin is longer, and thus an increase in NM myosin expression would increase the number of myosin attached to actin. This increase in the number of attached cross-bridges per unit time would increase force and vascular tone. This is supported by the results of a number of studies, which demonstrate that NM myosin contributes to the sustained phase of force maintenance in smooth muscle (Morano et al., 2000; Lofgren et al., 2003; Rhee et al., 2006; Yuen et al., 2009). Thus, the increase in NM myosin expression observed in PAH would contribute to the resting vasoconstriction that produces PAH.

### Treatment of PAH

Untreated patients with PAH have a median survival of 2.8 years, (Rich et al., 1987) but this has improved to over 7 years with the advancements of therapy (Benza et al., 2012). Patients in the World Health Organization (WHO) Functional Class I do not require any pharmacologic treatment, but do require monitoring. WHO Functional Class II, III, and IV require advanced therapy. As discussed above, a vasodilator challenge should be completed in patients who have confirmed PAH during a right heart catheterization, with symptoms warranting treatment. This challenge includes the administration of a short-acting vasodilator, either inhaled nitric oxide (10–20 ppm), intravenous epoprostenol (infusion rate of 1 to 2 ng/kg per minute increased by 2 ng/kg every minute until a drop in blood pressure is detected), or intravenous adenosine (50 mcg/kg per minute until maximal dose of 200–350 mcg/kg per minute). A positive response to the vasodilator challenge is defined as a mean pulmonary artery pressure (mPAP) decrease of ≥ 10 mmHg to a value ≤ 40 mmHg, and with a normal cardiac output. Hemodynamics must be closely monitored during a vasodilator challenge due to the potential hemodynamic collapse or abrupt development of pulmonary edema, particularly seen in patients with pulmonary veno-occlusive disease or pulmonary capillary hemangiomatosis. An acute vasodilator challenge should be avoided in patients with severely elevated filling pressures, to avoid such hemodynamic compromise (Barst et al., 2004).

The vasodilator challenge plays an important role in regards to therapy options for PH. Studies have illustrated that “responders” have survival benefits with long-term L-type CCB therapy. The acute pulmonary effect of the short-acting vasodilator therapy is thought to mimic the long term effect of CCB. Long-acting nifedipine or diltiazem can be utilized for treatment. It is important to emphasize that only 10–20% of patients with PAH have a positive vasodilatory challenge (Sitbon et al., 2005). The large majority of patients are considered “non-responders,” and are not candidates for CCB therapy (Barst et al., 2004). These patients receive other advanced therapies, such as phosphodiesterase 5 inhibitors (PDE5 inhibitors), which combine vasodilatory and antiproliferative properties. However, it is interesting to note that the “responders” are not treated initially with these advanced therapies; although it is reasonable to assume that these patients should also respond to therapies aimed at the nitric oxide pathway, such as PDE5 inhibitors, and guanylate cyclase activators. This raises the question of whether “responders” should also be initially treated with advanced therapies. Also, there is currently no way to predict who will be a responder. However, considering the information provided above, LZ+ MYPT1 expression could define a patient's response to NO; i.e., patients that maintain normal LZ+ MYPT1 expression would be “responders,” while those with a significant decrease in relative LZ+ MYPT1 expression would not respond to NO.

Advanced therapies are available to address the underlying process of PH, and treatments are aimed at the endothelin, nitric oxide, or prostacyclin pathways to reduce smooth muscle contractility (Figure 1). We are not aware an approved therapy for PAH aimed solely at reducing proliferation, and although some drugs have antiproliferative properties, their main mechanism of action is to produce a reduction in smooth muscle contractility. The categories for advanced PAH therapy include phosphodiesterase 5 inhibitors, sGC activators, endothelin receptor antagonists (ERAs), and prostacyclin pathway agonists. Phosphodiesterase 5 (PDE5) inhibitors include sildenafil, tadalafil, and vardenafil. As described above, abnormalities in the nitric oxide pathway lead to uninhibited vasoconstriction. Nitric oxide (NO) is synthesized from L-arginine by 3 NO synthases. Endothelial derived NO will diffuse into pulmonary arterial smooth muscle cells, and there, NO stimulates sGC to produce its second messenger cyclic guanosine monophosphate (cGMP) (Lincoln et al., 2001) and the increase in cGMP activates PKG, which in turn activates MLCP (Figure 1). cGMP is degraded by phosphodiesterases, and phosphodiesterase 5 is the predominant isoform expressed in the lung, and is increased in PAH (Jernigan and Resta, 2002). Inhibition of PDE5 increases cGMP and the subsequent activation of PKG signaling leads to vasodilatation (Lincoln et al., 2001; Galie et al., 2005). Studies have illustrated that patients with PAH benefit from PDE5 inhibitors, such as sildenafil, with overall subjective symptomatic and exercise capacity improvement, as well as objective improvement in 6 min walking distances and cardiopulmonary hemodynamic parameters (Galie et al., 2005). The 6 min walking distance is often used as a primary endpoint for studies involving PAH, and this serves as an independent predictor of death and correlates with survival. Although, 6 min walking distances are improved with the use of sildenafil and other PDE5 inhibitors, there has been no clear improvement in overall mortality (Galie et al., 2005).

Guanylate cyclase stimulants also act on the nitric oxide pathway (Figure 1) to increase vasoreactivity and combat the chronic vasoconstriction associated with PAH. sGC is a nitric oxide receptor, and stimulants or activators act via two mechanisms: to increase the sensitivity of sGC to endogenous nitric oxide and directly stimulate the guanylate cyclase receptor, independent of nitric oxide availability, to simulate the activity of nitric oxide. Riociguat is member of the therapeutic class of sGC stimulators, and patients with PAH also have proven benefit from riociguat. Ghofrani et al. (2013b) illustrated that riociguat significantly improved overall exercise capacity in patients with PAH. There were also notable improvements in pulmonary hemodynamics, WHO functional class, and 6 min walking distances (Ghofrani et al., 2013b). Surprisingly, Riociguat has also been shown to offer benefit in patients with CTEPH or Group 4 PH (Ghofrani et al., 2013a).

ERAs are another subset of advanced therapy for PAH. Endothelin-1 is a potent vasoconstrictor, which acts through 2 receptors, including ET_A_ and ET_B_. Both receptors are found in pulmonary artery smooth muscle cells (PASMCs) and help to mediate vasoconstriction. Of note, ET_B_ mediates NO and prostacyclin release that actually result in vasodilation, but this is only on endothelial cells, not in PASMCs (Archer et al., 2010). Endothelin agonists activate the Rho/ROK pathway to inhibit MLCP and also have downstream effects including activation of CPI-17, which will also inhibit MLCP (Figure 1). PAH patients do exhibit increased levels of endothelin-1 in the lung. ERAs, such as ambrisentan, bosentan, and macitentan inhibit the vasoconstrictive properties of endothelin-1. Bosentan and macitentan are non-selective ERAs, while ambrisentan is a selective receptor antagonist for endothelin receptor A. The main side effects for all of these medications are hepatotoxicity and peripheral edema. Ambrisentan, the oral selective ET_A_ receptor antagonist is thought to be the least hepatotoxic of these agents. All ERAs have demonstrated improvements in exercise capacity, symptoms, and pulmonary hemodynamics (McLaughlin et al., 2015).

Fasudil, a Rho kinase inhibitor is a newer therapy for PAH. The inhibition of Rho kinase has acute and prolonged changes, including vasorelaxation of the pulmonary artery and reduction of structural remodeling by the inhibition of smooth muscle cell proliferation. Studies have shown that monocrotaline associated PAH is improved with fasudil, which results in a reduction in RV dilatation and hypertrophy (Oka et al., 2007). Fasudil also has been shown to restore or increase endothelial NO synthase expression and cGMP levels, resulting in vasodilation (Mouchaers et al., 2010).

The final category for advanced therapy in PAH is the prostacyclin pathway agonists, which increase endogenous prostacyclin production with the use of exogenous prostanoids (Archer et al., 2010; McLaughlin et al., 2015). Pulmonary vasoconstriction and endothelial dysfunction in PAH causes reduced prostacyclin synthase expression, therefore resulting in decreased prostacyclin, which is a vasodilator with antiproliferative effects. The signaling pathway for vasodilatation begins with fatty acid cyclooxygenase, which will convert arachidonic acid to prostaglandin H_2_, the substrate for prostaglandin I_2_ or prostacyclin. Prostacyclin stimulates adenylate cyclase, which catalyzes the conversion of ATP to cAMP, in turn activating PKA (Figure 1). The activation of PKA reduces intracellular Ca^2+^ and inhibits MLCK (Miller et al., 1983), which leads to vasodilatation. The administration of prostanoids produces both vasodilation and reduction in proliferation. Drugs in this category include intravenous prostacyclin or epoprostenol, synthetic prostacyclin in the form of inhaled, intravenous, or subcutaneous treprostinil or inhaled iloprost, and the oral prostacyclin receptor agonists, such as selexipag. Intravenous epoprostenol has been the most closely studied, and studies have illustrated significant improvement in survival and pulmonary hemodynamics (Barst et al., 1994). Originally and until 2001, epoprostenol was the only treatment available for PAH, specifically used for bridge to transplantation.

In addition to the classic therapies, combination therapy is also being utilized for severe PAH. Previously, the standard of care was initial single-agent therapy with the sequential addition of therapy when patients failed to show significant improvement. The BREATHE-2 trial showed that combination therapy with bosentan plus epoprostenol vs. epoprostenol alone trended toward improvement in hemodynamics; however the difference was non-significant (Humbert et al., 2004). In the AMBITION trial, which was a large-scale prospective double-blinded study, patients were randomized to combination therapy with ambrisentan and tadalafil or monotherapy with either ambrisentan or tadalafil. The primary endpoint was the time to first clinical failure event, either death, hospitalization for worsening PAH, or a significant increase in symptoms, and the risk of clinical failure was decreased in patients treated with combination therapy (Galie et al., 2015). Sitbon et al. (2016) recently demonstrated that initial combination therapy with an endothelin receptor antagonist plus a PDE5 inhibitor resulted in improved prognostic indicators, including a longer 6 min walking distance, lower right atrial pressure, and improved cardiac index (Sitbon et al., 2016). Therefore, in the future, there may be a trend toward initial dual oral therapy with a combined ERA and PDE5 inhibitor.

The choice of therapy and the treatment plan should be individualized to the needs of the patient. Although, treatments for PAH are designed to attack one of the signaling pathways important for the regulation of vascular tone (Figure 1), there is little data regarding the selection of one drug over another in individual patients, and cost surely must influence selection. If CCBs do not improve hemodynamics and symptoms in the “responders,” most would initiate therapy with a PDE5 inhibitor (McLaughlin et al., 2015). Similarly, PDE5 inhibitors are generally considered as an initial therapy in “non-responders.” However, the decrease in sensitivity to cGMP/NO could result from a decrease in MYPT1 LZ+ expression. Increasing cGMP and moving up the dose response relationship for NO/cGMP mediated relaxation could produce a decrease in tone, and therefore, one could argue that increasing cGMP in patients with PAH could produce some degree of vasodilatory response. However, this is purely conjecture, as no data specifically addresses this point. Nonetheless, since NO mediated vasodilatation is a fundamental response of the vasculature (Furchgott, 1999), we would suggest that either PDE5 or sGC activators should be primary therapy in all patients with PAH. If there is little or no sustained improvement in functional class, we would argue that attacking both NO/cGMP signaling to increase smooth muscle relaxation, and inhibiting smooth muscle contractility with prostacyclin analogs, ET-1 antagonists, or Rho kinase inhibitors should be considered as the next step in therapy.

It is also important to consider the severity of PAH, which can be determined by symptoms/WHO Functional Class, echocardiographic and hemodynamic parameters, and co-morbidities. Higher risk features and markers of worse outcomes include WHO Functional class IV, a 6 min walking distance of <300 m, a pericardial effusion on transthoracic echocardiogram, a right atrial pressure >15 mmHg, a cardiac index ≤ 2 l/min/m^2^, or a RVEF of <35% on cardiac MR. The first steps in determining treatment include proper diagnosis with transthoracic echocardiography, pulmonary function testing, V/Q scan, laboratory testing, and invasive hemodynamic testing to confirm the true presence of PAH and rule out another cause of PAH. The next question to ask is whether the patient had a positive response to acute pulmonary vasodilator testing. Current guidelines recommend that all patients with a positive response be treated with CCBs. However, it could be argued that those patients who respond to acute vasodilators would also benefit from initial therapy with PAH advanced therapies, particularly given only a small percentage of patients (McLaughlin et al., 2015) respond well over time to chronic CCB therapy. As mentioned above, in more severe cases of PAH, initial combination therapy may be beneficial. In cases of less severe PAH, sequential therapy may be most appropriate. However, which pathway should be chosen to address first? Given the decreased response to nitric oxide as an underlying mechanism for PAH, addressing the nitric oxide pathway could be a first-line strategy for treatment. As mentioned above, the two outlined methods for increasing NO responsiveness focus on cGMP. PDE-5 inhibitors decrease cGMP degradation, and the sGC stimulators increase cGMP production. Oral endothelin antagonists could then sequentially be added for combination therapy. Intravenous prostacyclins should be utilized first for severe cases of PAH. For end-stage disease, lung transplantation is often the only option.

### Novel approaches to treatments

Every current therapy for PAH is designed to decrease contractility of the pulmonary vascular smooth muscle, and it is important to also explore novel approaches to PAH treatment. Each of the current therapies attack a signaling pathway that regulates contractility, and all of the signaling pathways converge to inhibit the interaction of actin with myosin, either by inhibiting MCLK or activating MLCP (Figure 1). It is unlikely that only a single signaling pathway is responsible for the pathogenesis of PAH in each patient. More likely, there is a variable contribution from each pathway that results in PAH, which is consistent with the benefit of combination therapy. This hypothesis would suggest that therapies for PAH should focus on increasing the activity of MLCP and/or decreasing the activity of MLCK, which should result in a decrease in pulmonary vascular tone (Gong et al., 1992). Gene therapy is beginning to emerge as a treatment for both cystic fibrosis (Griesenbach et al., 2004) and heart failure, (Jessup et al., 2011; Chung et al., 2015; Greenberg et al., 2016; Hulot et al., 2016) with both viral and non-viral methods of gene transfer. Thus, one could consider using gene therapy to tune the activity of MLCK and/or MLCP as a treatment for PAH. Investigators have demonstrated that the soybean isoform of calmodulin (SCaM4) binds Ca^2+^, binds tightly to MLCK, but does not activate MLCK (Lee et al., 2000). Further, when exchanged into skinned smooth muscle strips, SCaM4 inhibits Ca^2+^ activated force (Van Lierop et al., 2002), suggesting the expression of SCaM4 in pulmonary vascular smooth muscle would inhibit the activation of MLCK and result in a decrease in MLC phosphorylation and force, which would reduce pulmonary pressures. These data suggest that gene therapy carrying SCaM4 could be a novel translational approach for the treatment of PAH. Therapies designed to tune the activities of MLCK and MLCP potentially offer hope for improving morbidity and mortality in patients with this disease.

In PAH, NM myosin expression is also increased (Packer et al., 1998; Konik et al., 2013). As described above, the slow kinetics of NM myosin (Kovacs et al., 2003; Wang et al., 2003) will increase the time cross-bridges are attached to actin. Therefore, an increase in NM myosin expression would increase the number of attached cross-bridges per unit time, which increase smooth muscle tone. Others have demonstrated that NM myosin participates in the force maintenance phase of smooth muscle contraction (Morano et al., 2000; Lofgren et al., 2003; Rhee et al., 2006; Yuen et al., 2009); and thus in PAH, the increase in the expression of NM myosin in pulmonary smooth muscle (Packer et al., 1998; Konik et al., 2013) would produce an increase in PVR. NM myosin is regulated by phosphorylation of the NM myosin RLC (Sellers and Adelstein, 1985) and data suggest that during G-protein coupled agonist activation, NM RLC phosphorylation is dependent on the activation of MLCK and Rho kinase (Yuen et al., 2009). These data suggest that Rho kinase inhibition would preferentially decrease the activation of NM myosin, and for PAH, potentially be more beneficial than therapies designed to inhibit other signaling pathways. However, the ultimate goal of therapy would be to inhibit the interaction between actin and myosin, either smooth muscle myosin or non-muscle myosin.

Also, as mentioned above, in PAH, LZ+ MYPT1 expression is decreased (Singh et al., 2011; Konik et al., 2013). LZ+ MYPT1 expression has been demonstrated to fall in both hypoxia (Singh et al., 2011) and PAH (Packer et al., 1998; Konik et al., 2013). In the pulmonary vasculature, a decrease in LZ+ MYPT1 expression would both decrease the sensitivity to NO mediated vasodilatation (Huang et al., 2004; Yuen et al., 2014) as well increase the sensitivity to Rho kinase mediated vasoconstriction (Lin and Brozovich, 2016), which would produce an increase in PVR. These data suggest that LZ+ MYTPT1 expression would predict not only a response to NO, but thus, also patient prognosis; and that gene therapy designed to increase LZ+ MYPT1 expression would be another novel therapy for PAH. There are other delivery strategies that could be used to target delivery of sCaM4 and/or LZ+ MYPT1 to the pulmonary vasculature. Aerosol delivery of AAV9 results in robust infection of the pulmonary vasculature (Kataoka et al., 2013; Gubrij et al., 2014). Also the lipid nanoparticle, Star:Star-mPEG, is effective in specifically delivering proteins to the pulmonary vasculature (McLendon et al., 2015).

## Summary/conclusions

Both structural changes and abnormalities at the level of the regulation of smooth muscle contractility contribute to the mechanism producing PAH. However, all drugs approved for treatment of this disease focus on decreasing smooth muscle contractility, and we have outlined several strategies to decrease pulmonary vascular tone, and possibly improve pulmonary hemodynamics. Further investigation is required to identify patients that will respond to a vasodilator challenge and develop therapies that will improve both symptoms and the prognosis of patients with PAH.

## Author contributions

ML, JD, and FB all contributed to both the conception and writing of the manuscript.

### Conflict of interest statement

The authors declare that the research was conducted in the absence of any commercial or financial relationships that could be construed as a potential conflict of interest.
